# On the Internal Use of Chloroform for the Relief of Biliary Calculi

**Published:** 1873-11-01

**Authors:** A. Gaslin

**Affiliations:** Oregon, Wis.


### ON THE INTERNAL USE OF CHLOROFORM FOR THE RELIEF OF BILIARY CALCULI.

BY A. GASLIN, M.D., OREGON, WIS.

In *Braithewait*, for July 1870, Dr. John Barclay says: "Seeing some reference in a contemporary periodical, to the proposed use of chloral as a solvent of biliary calculi, I crave a space to state that I have met with very great success from the internal administration of chloroform in that disease.

"I first used it in 1861, in the case of a clergyman aged fifty-eight. He had suffered for twenty-three years from gall-stones; the peculiar pain, jaundice, with subsequent discharge, by stool, of the calculi, coming on so suddenly, and without warning, as seriously and frequently to interfere with his duties.

"Just then, writing on alcohol, I had been studying the experiments of Lallemand, and others, on the existence of alcohol unchanged in the blood.

"Knowing that ethers are solvents of cholesterine, I ventured, on his third attack in that year, to prescribe chloroform in doses of two or three drops, three or four times a day, on the chance of its reaching the calculi through the blood. To his surprise, and my gratification, pain, tenderness, distension, and jaundice, disappeared together; and in the eight years since elapsed, he has never had another attack. He keeps a bottle of "chloric ether" by him, for occasional use. I have found it to give invariable and permanent relief in many instances since. The theory of thus dissolving the calculi in situ, followed by the disappearance of the symptoms, leads to a deduction, that may be legitimate, that relief was obtained by their being so dissolved."

Shortly after this article came to my notice, I had occasion to try it in two cases having many of the symptoms of biliary calculi, yet not possessing the positive evidence of finding calculi in the stools. Both cases were promptly relieved. I will give a report of two cases where all the evidence is positive:

Case I.—September 17th, 1872, I was called to see Mrs. B——, aged fifty-eight years; had been a very stout woman, of very active habits; has done a great deal of hard work. I had met her two years before, when she was quite fleshy and robust; but at this visit I would scarcely have known her, she was so reduced in flesh: skin very yellow, and a general haggard expression, which, to me, betokened an early dissolution, unless I could give her relief.

I obtained from her this history of her case: She said for the past ten years she had suffered occasional attacks once or twice a year, her physician treating her for bilious colic. The preceding February she had been taken down much more violently than ever before, and the attacks recurring every two or three weeks since that time.

She would be taken with a chill, lasting her twenty-four to thirty-six hours; great pain in right side, over liver, extending to stomach; vomiting and nausea all the time. After chill would pass off, a hot, dry skin, enlargement of liver, with great tenderness over liver and stomach, jaundiced hue of eyes and skin, would follow. Then she would be better for a week or two, when the same thing would occur, except each attack was more severe. She had been treated by an old physician (probably not the best) for engorgement of liver, for inflamation of liver, etc., etc., with calomel quinine, blisters, and divers and sundry other remedies.

She was profusely ptyalized, and had been for two weeks, with the foolish hope of geting her liver active; pulse feeble and frequent; had taken no nourishment for days, nor could she take any, on account of constant nausea. I strongly suspected gall-stone as her trouble, and ordered a stimulating enema to be given, with orders to wash the stools and look for gall-stones; also gave her a mouth-wash of carbolic acid and chlorate potass., internally, and stopped all other drugs.

September 18th: visited her to-day; in washing the stools, as directed, found two gall-stones, each about two-thirds as large as grains of corn; irregular in shape. Now, I had plain sailing before me—positive evidence. I gave her an ounce mixture, equal parts of chloroform and alcohol, and directed her to take eight drops, three times a day; continued mouth-wash; and to take beef-tea, if she could retain it.

September 20th: better; stomach retentive; takes nourishment, and relishes it; mouth much improved; liver less tender, and not so much enlarged; skin and eyes clearing up.

September 24th: improving in every respect.

September 28th: improving rapidly; skin almost natural in color; appetite very good; no pain, tenderness or enlargement of liver. She has taken no medicine but the mixture of chloroform and alcohol; and at this time, October 1st, has not had a recurrence of her trouble. She keeps her medicine by her, and takes a dose occasionally, not oftener than once or twice a week; and she has regained her usual flesh and robustness, and does her usual amount of hard work.

CASE II is that of a lady I have never seen. She wrote a letter to a brother living in this place, giving him a history of her sickness. She stated she had been suffering for eight months with pain in right side, in region of liver, with frequent attacks of chill, with great increase of pain, followed with jaundice.

Finally, a tumor began to form, and pointed just below the cartilages of ribs. With repeated blistering, this tumor opened and discharged large quantities of gall-stones. At the time of writing she said over one hundred had been discharged; said the fistulous opening would not heal on account of their passage. I was shown the letter, and at once recommended her to use the mixture of chloroform and alcohol, same as first case, and stop all other medicines.

The fifth day after she commenced taking the remedy, she passed the last gall-stone, and in a short time the fistulous opening closed, and her health has been good ever since. May we not reasonably conclude, with Dr. John Barclay, that "the theory of thus dissolving the calculi insitu, followed by the disappearance of the symptoms, leads to a deduction that may be legitimate, that relief was obtained by their being so dissolved?"

